# Molecular Mechanism of Action of HOCl from Neutral-pH Electrolysed Oxidising Water Against *Candida albicans*

**DOI:** 10.3390/jof11110761

**Published:** 2025-10-23

**Authors:** Chitra S. Krishnan, Trudy J. Milne, Geoffrey R. Tompkins, Richard D. Cannon, Erwin Lamping

**Affiliations:** Sir John Walsh Research Institute, Faculty of Dentistry, University of Otago, Dunedin 9016, New Zealand; chitra.krishnan@otago.ac.nz (C.S.K.); trudy.milne@otago.ac.nz (T.J.M.); geoffrey.tompkins@otago.ac.nz (G.R.T.); erwin.lamping@otago.ac.nz (E.L.)

**Keywords:** super-oxidised water, electrochemically activated water, denture disinfection, hypochlorous acid, molecular response, RNA-sequencing, *Candida albicans*, *PRN1*, *HSP21*

## Abstract

Chemical disinfection of removable acrylic dental prostheses minimizes the risk of denture stomatitis caused by the opportunistic fungal pathogen *Candida albicans*. We previously reported that neutral-pH electrolysed oxidising water (EOW), a hypochlorous acid (HOCl)-based biocide, is effective at inhibiting *C. albicans* biofilm formation on denture resins. Knowledge about the mechanism of action of EOW on *C. albicans* is lacking. This study investigated the molecular mechanism of action of neutral-pH EOW against *C. albicans* cells that were incubated with sub-inhibitory concentrations of EOW-HOCl (treatment with 0.125× MIC_90_ EOW-HOCl (15 µM; T0.125) or treatment with 0.5× MIC_90_ EOW-HOCl (59 µM; T0.5)). RNA-sequencing (RNA-seq) was used to identify differentially expressed genes (DEGs) which were validated by qRT-PCR. Ninety-five DEGs were identified between the treated and untreated cells after a 60 min exposure. A moderate sub-inhibitory EOW-HOCl concentration (T0.125) caused significant upregulation (log_2_ fold change > +2) of genes responsive to oxidative stress (*EBP1*, *GAP6*, *PRN1*, *HSP21*), weak organic acid stress (*PRN1*), and heat-shock (*HSP21*). A higher sub-inhibitory concentration (T0.5) caused a significant downregulation of most DEGs (notably, −1.9 to −3 log_2_ fold reduction in *SUT1*, *HNM3*, *STP4* expression), cessation of growth, and an upregulation of genes involved in ammonia transport, carbohydrate metabolism, and the unfolded protein and apoptotic response pathways (*ATO2*, *IRE1*). Our findings reveal *HSP21* and *PRN1* to be possible key players in protecting *C. albicans* cells against HOCl, a natural biocide of the innate immune system.

## 1. Introduction

Investigations of the effects of hypochlorous acid (HOCl) and hypochlorite anions (ClO^−^) on microbial cells have predominantly focussed on the effects of sodium hypochlorite (bleach, NaOCl) on bacteria [1,2,3,4,5,6,7,8,9]. The active chlorine species in NaOCl solutions (pH~11–13) is the weakly oxidising ClO^−^. However, NaOCl at neutral pH has been used to study the effects of HOCl on microbial cells. HOCl is a weak acid (p*K*_a_ 7.53) that exists as a mixture of the acid and the partially dissociated proton and hypochlorite anion (HOCl/H^+^ plus ClO^−^) at near-neutral pH [10]. The antibacterial action of bleach at neutral or near-neutral pH is largely due to the ability of HOCl to aggregate bacterial proteins. Bacteria have mechanisms that respond to this protein aggregation. Activation of the redox-regulated chaperone Hsp33 has been identified as a potential defence mechanism of *Escherichia coli* in response to the HOCl-induced aggregation of essential proteins [8]. In addition, HOCl was reported to induce enzymes of the oxidative stress response pathway, including catalases, peroxidases, and superoxide dismutase in Gram-negative bacteria [11]. HOCl was also found to activate other protein chaperones, DNA and protein repair systems, and methionine sulfoxide reductases (MSRs), increase membrane hydrophobicity, and reduce membrane permeability and the expression of porins in Gram-negative bacteria [11]. Thus, the lethal effect of HOCl on bacterial cells is thought to be due to oxidative burst-induced stress [4]. Some Gram-negative bacteria can also adapt to sublethal HOCl concentrations by adopting a ‘viable but non-culturable’ (VBNC) state, and by biofilm formation [11].

Knowledge of the mechanism of HOCl-triggered death in yeast is limited, although oxidative damage is possibly key in *Saccharomyces cerevisiae* [12,13]. However, a recent study found that HOCl induces a rather different transcriptional response in *C. albicans* than the oxidant H_2_O_2_ [14]. Both *C. albicans* and *S. cerevisiae* have been studied extensively to understand yeast responses to various important stress conditions. Such investigations have centred on macrophage/neutrophil phagocyte-survival models to understand the pathways triggered by reactive oxygen species (ROS) and the response to H_2_O_2_-, weak-acid-, and NaOCl-induced stress [10,12,13,14,15,16,17,18,19,20,21,22,23,24]. *C. albicans* responds to oxidative stress by upregulating enzymes involved in the removal of H_2_O_2_ and free radicals, such as superoxide dismutase (*SOD1*, *SOD2*, *SOD5*), enzymes of the thioredoxin (*TSA1*, *TRX1*, *TRR1*) pathway, and genes encoding the glutathione/glutaredoxin (*GPX1*, *GSH1*) system [17,25]. The *CAT1* (also known as *CTA1* [26]) gene, which encodes the key antioxidant enzyme catalase, is another *C. albicans* gene that responds to osmotic, oxidative, and heavy metal stress [17]. Osmotic (NaCl), oxidative (H_2_O_2_), and heavy metal (CdSO_4_) stresses significantly upregulate similar core response genes, probably regulated by the fungal-specific Hog1-SAPK (stress-activated protein kinase) pathway. Other pathways, such as the Cap1 (AP-1 bZIP transcription factor) pathway, either acting alone or in parallel, contribute to the transcriptional response to oxidative stress [17,18]. The small heat shock protein Hsp21 of *C. albicans* has been implicated in protecting cells against neutrophil attack and oxidative stress (induced by menadione). Hsp21 is a virulence factor that enables *C. albicans* to adapt to elevated temperatures via the Cek1 kinase stress-response pathway [22]. Heat-shock proteins (HSPs) help organisms adapt to many stress conditions such as elevated growth temperatures, oxidative stress, or starvation [27]. There are five major HSP families, the rather highly conserved ATP-dependent Hsp100s, Hsp90s, Hsp70s, and Hsp60s families and the less conserved ATP-independent family of small HSPs ranging in size from 12 to 42 kDa [28]. *HSP21* homologs are only found in some other *Candida* CTG clade species [22]. The relatively recently discovered pirin-like protein, Prn1 is possibly another important factor involved in protecting *C. albicans* cells from oxidative and weak organic acid stress [15,29]. Although *S. cerevisiae* has been used as a model yeast to study stress-response mechanisms, the responses of *C. albicans* to thermal, osmotic, and oxidative (H_2_O_2_) stress is quite unique, with no significant induction of genes common to the three main stress response pathways in *S. cerevisiae*. Apparently, *C. albicans* lacks the strong general stress response pathway exhibited by *S. cerevisiae* [18].

**Figure 1:**
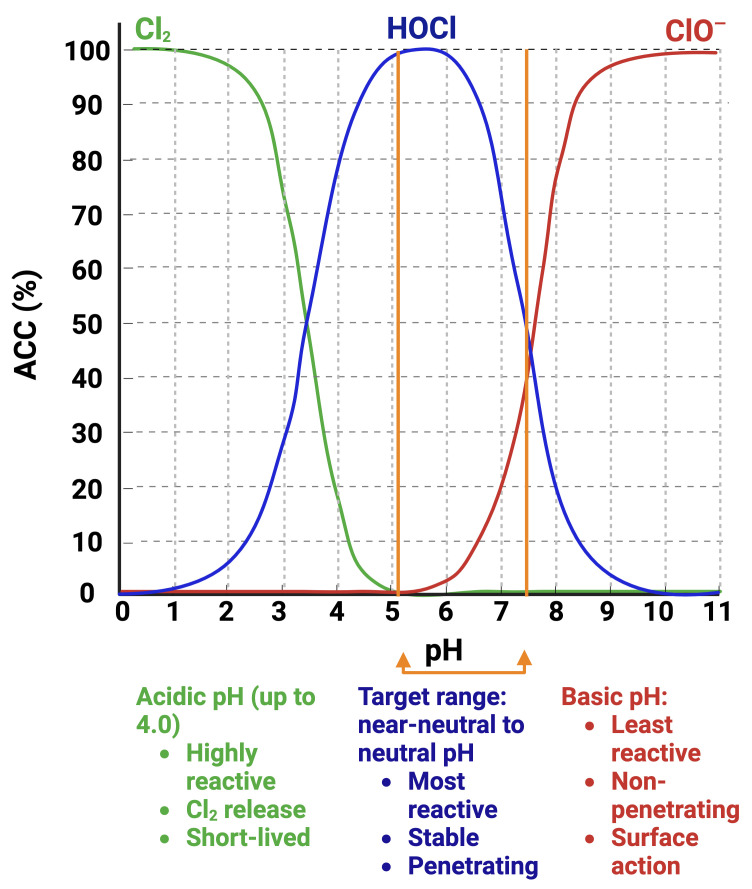
Interrelationship of pH and chlorine species (available chlorine content, ACC) present in EOW. The predominant chlorine species (Cl_2_, HOCl, or ClO^−^) is pH-dependent which determines the solution stability and activity [31,32]. Created with BioRender.com.

Response to HOCl in yeasts relates to its oxidative activity and the production of ROS typically found in NaOCl solutions [10,12,20,24]. The active chlorine species in NaOCl solution at pH 13–13.5, is the ClO^−^ anion [30] (Figure 1). Thus, to study the effects of HOCl, pH adjustments are necessary to bring the NaOCl solution into the moderately acidic-to-neutral-pH range in which HOCl predominates. In moderately acidic-to-neutral-pH EOW, the active component is HOCl (Figure 1), which makes this a suitable agent to study the molecular mechanism of action of HOCl against *C. albicans* and related yeast species.

Ready-to-use (RTU), neutral-pH EOW is an effective, surface and medical equipment disinfectant and sanitizer that lacks the toxicity associated with NaOCl (bleach) [33]. However, its mechanism of action against *C. albicans* is not well understood. The aim of this study was to explore the molecular mechanism of action of sub-growth-inhibitory concentrations of neutral-pH EOW-HOCl on *C. albicans* by analysing the transcriptional response via mRNA sequencing and RT-qPCR. We hypothesized that sub-growth-inhibitory concentrations of EOW (HOCl) would elicit a change in the expression of genes involved in the *CAT1*, *SOD1*, and *TRR1* pathways, similar to the response to ROS and HOCl generated by neutrophils [20,25].

## 2. Materials and Methods

### 2.1. Determination of the Minimum Growth Inhibitory Concentration of EOW-HOCl Against C. albicans SC5314

MIC values refer to the undissociated HOCl concentration in the EOW (MIC_90_ EOW-HOCl). The [HOCl] of bottled EOW (Envirolyte, Auckland, New Zealand) was determined by UV–Vis spectrophotometry [34]. MIC assays were performed in 96-well microtitre plates using a method adapted from the EUCAST EDef7.1 2008 protocol [35] to determine the MIC_90_ of EOW-HOCl against *C. albicans* SC5314 at the higher cell densities required to generate sufficient RNA for RNA sequencing. *C. albicans* SC5314 (Molecular Biosciences Laboratory, Faculty of Dentistry, University of Otago, New Zealand) was propagated on sabouraud dextrose agar (SDA; Fort Richard, Auckland, New Zealand). Individual colonies were suspended in sterile ddH_2_O, and cell densities were adjusted to 10^7^ cfu/mL (OD_540_ = 1.0) and 10^8^ cfu/mL (OD_540_ = 10.0). Aliquots (10 µL) were added to microtitre wells containing 90 µL two-fold serial dilutions of EOW (80% to 0.3%) in sterile ddH_2_O, including a 90 µL no-EOW control, allowed to react for 1 and 5 min, and quenched by the addition of 100 µL 2× RPMI-1640-2%G medium (RPMI-1640; Sigma-Aldrich, Merck (Darmstadt, Germany), containing 0.165 mM MOPS buffer, Sigma-Aldrich, Merck (Darmstadt, Germany), and 2% glucose, pH 7.0). Cell suspensions were incubated statically at 37 °C for 20 h and the OD_540_ determined with a plate reader (Synergy 2, Bio Tek Instruments, Winooski, VT, USA). Wells with the lowest EOW concentration that had an OD_540_ reading at least 90% lower than the non-treated control wells indicated the MIC_90_ and the corresponding HOCl concentration was calculated. In other experiments, MIC assays were performed in a similar fashion with an inoculum of 10^8^ cfu/mL, but with YNBG medium (YNB media w/o amino acids, Formedium Ltd., Hunstanton, UK, with 2% glucose). Parallel assays involved adding 10 µL of the inoculum to 90 µL of EOW dilutions pre-mixed with 100 µL 2× YNBG medium to rule out quenching effects of YNBG.

### 2.2. Isolation of Total RNA from C. albicans SC5314 Cells

*C. albicans* SC5314 was grown in YNBG (250 mL) inoculated to an OD_540_ of 0.05 with cells from a 16 h, 37 °C, YNBG starter culture. The culture was incubated at 37 °C with shaking until the OD_540_ was between 1.0 to 2.0 at which point the cultures were treated (T) with sub-growth-inhibitory concentrations of EOW-HOCl for different amounts of time; control cultures were not treated with EOW. Cells from 100 mL of the culture were harvested by filtration through a glass fibre filter No. 6 (Schleicher & Schuell BioScience GmbH, Dassel, Germany) using a vacuum manifold and washed once with 5 mL ice-cold distilled water [36]. The ‘cell cake’ was scraped off the filter with a scalpel, quickly transferred into a 1.5 mL microcentrifuge tube, snap frozen in liquid nitrogen and stored at −80 °C. Total RNA was extracted from frozen cell pellets (∼100 mg wet weight) using a hot-phenol extraction protocol [36]. Frozen cell pellets were placed in 15 mL Corex tubes containing a mixture of 1 mL acid phenol (saturated with SAB buffer; 50 mM sodium acetate, 10 mM EDTA, pH 5.0), 2 mL SAB buffer, 100 μL 10% SDS, and ∼1 g zirconia beads (0.5 mm diameter; BioSpec Products, Bartlesville, OK, USA) and heated to 65 °C in a water bath. The cells were broken by five cycles of 30 s vortexing (max speed 2500 rpm; IKA Vortex, Genius 3, Selangor Malaysia) with 1.5 min incubation at 65 °C between each cycle. Liquid phases were separated by centrifugation at 10,000× *g* for 10 min and traces of phenol in the ∼2 mL upper phase removed by two rounds of extraction with ~1/3 volume of chloroform. Total RNA remaining in the supernatant was precipitated with 1/10 volume of 3 M sodium acetate, pH 6.0, and 3 volumes of 100% EtOH and harvested by centrifugation at 30,600× *g* for 30 min at 4 °C. The RNA pellets were then washed with 1 mL 70% EtOH to remove traces of salt, air dried for 10 min, and dissolved in 200 μL RNAse-free H_2_O. Traces of gDNA were removed by DNase treatment (PureLink DNase kit, Invitrogen Inc., Carlsbad, CA, USA) followed by ethanol precipitation and resuspending the pellets in 0.5 mL RNAse-free H_2_O. RNA purity and concentrations were determined with a NanoDrop UV–Vis spectrophotometer (GE Healthcare, Amersham, UK), adjusted with RNAse-free H_2_O to 0.5 µg/µL, and the quantity and integrity of the total RNA extracts were confirmed by gel electrophoresis.

### 2.3. cDNA Template Synthesis

First strand cDNA was synthesised from 2 μg total RNA in a 20 µL reaction volume using the SuperScript IV VILO Master Mix (Invitrogen Inc., Carlsbad, CA, USA) following the manufacturer’s instructions. Control reactions without reverse transcriptase (minus RT controls; −RT) were included for each sample to confirm that the samples were not contaminated with residual gDNA.

### 2.4. RT-qPCR Assays

Forward and reverse primers to amplify genes of interest (Table S1) were custom-synthesised (Sigma-Aldrich, Merck, Darmstadt, Germany) and are listed in Table 1. Primers were designed to amplify similar size (~125 bp) amplicons for each gene. Amplification efficiencies for each qPCR primer pair were determined using dilutions of first strand cDNA templates starting with 10, 5, 4.2, and 3.8 ng total RNA (Figure S1). RT-qPCRs were performed in a reaction volume of 10 µL and contained 1× PowerTrack SYBR Green Master Mix (Applied Biosystems Inc., Foster City, CA, USA), 0.4 µM of each primer, and one of the cDNA dilutions. Reactions were carried out in a QuantStudio 6 Flex Real-Time PCR System (Applied Biosystems Inc., Foster City, CA, USA). Thermal cycling steps were 95 °C for 20 s, followed by 40 cycles of 95 °C for 3 s and 60 °C for 30 s, followed by a melt (dissociation) cycle to assess the quality of the amplification products. Negative controls (no template and −RT) were included. There were two technical replicates within each RT-qPCR assay, and there were 3–6 independent repeats of each assay. The mean C_q_ values from all the repeat assays were used for gene expression analyses. mRNA transcript levels (2^−∆Cq^) were normalised to the housekeeping gene *TDH3*. The fold-change of mRNA levels in the treated group relative to the control was calculated with the ∆∆C_q_ method (2^−∆∆Cq^) [37]. The mean fold-change of mRNA levels (±SD) was plotted on X-Y bar graphs using GraphPad PRISM, v9.0.

### 2.5. RNA Sequencing

Total RNA samples (5 µg) from the C60, T60 0.125, and T60 0.5 groups (three independent replicates per group) were individually packaged as dry samples in RNA stabilization tubes (Genewiz, Azenta Life Sciences, Suzhou, China) following the manufacturer’s instructions for next generation sequencing (NGS) of mRNA using the Illumina sequencing platform (Illumina Novaseq6000, San Diego, CA, USA). RNA sequencing was conducted by Genewiz as described in Supplementary Materials and Figure S2.

Adapter sequences, PCR primers, or fragments thereof, and sequences with quality values < 20 were removed and raw sequence data (.bcl files) generated were converted into fastq files and filtered with Cutadapt (v1.9.1, phred cutoff: 20, error rate: 0.1, adapter overlap: 1 bp, minimum length: 75, proportion of N: 0.1). Sequencing reads that were <75 bases were also removed by this process to leave only high quality clean data [38,39]. The filtered transcripts were aligned against the reference genome sequence of *C. albicans* SC5314 (Assembly 22, ASM18296v3, available from: https://www.ncbi.nlm.nih.gov/genome/?term=ASM18296v3, accessed on 1 June 2023) using Hisat2 (v2.0.1) [40] with default parameters. Transcripts in fasta format were converted from known General Feature Format (gff) annotation files and indexed; gene and isoform expression levels from the pair-end clean data were estimated using HTSeq, (v0.6.1) [41]. Gene expression was calculated as FPKM (fragments per kilobase per million reads), based on read counts from HT-seq (v0.6.1) [42]:

Statistically significant differences of gene-specific fold change (log_2_ transformation) were generated with the untreated group serving as the control. Differential expression analysis was performed using the DESeq2 Bioconductor package (v1.6.3) [43] based on a model with negative binomial distribution; *p* (adjusted) of genes was set at <0.05 to detect differentially expressed genes (DEGs). The results from the DESeq2 analysis were further analysed to identify genes with significant differential expression according to the criteria of fold change greater than 2 (up- or down-regulated) and q value (false discovery rate, *p* adj < 0.05). These log_2_ fold-change values were used to plot column-clustered heatmaps using the WGCNA R package(v2.10.0): http://www.genetics.ucla.edu/labs/horvath/CoexpressionNetwork/Rpackages/WGCNA, accessed on 15 June 2023 [44]. The correlation between fold-changes in gene expression determined by qRT-PCR and by RNAseq was determined using the Pearson’s correlation coefficient test.

GO (Gene Ontology Consortium Database; [45]) functional enrichment analysis was performed to classify the biological functions of significant DEGs (GOSeq, v1.34.1) [46]. Gene length and read count biases were included in the GO analysis using a filtering threshold for over-represented sequences by applying *p* < 0.05. Directed Acyclic Graph (DAG) graphical representations of the results of enrichment analysis of the differentially expressed genes were obtained using TopGO (v2.18.0). In-house scripts were used in pathway enrichment analysis based on KEGG pathway units [47] from the public pathway database, KEGG (Kyoto Encyclopedia of Genes and Genomes), using a hypergeometric test to find the pathways of the differentially expressed genes that were significantly enriched compared to the transcriptome background.

## 3. Results

### 3.1. MIC_90_ of EOW-HOCl at High Inoculum Densities and Culture Volumes

#### Microtitre MICs

**Figure 2:**
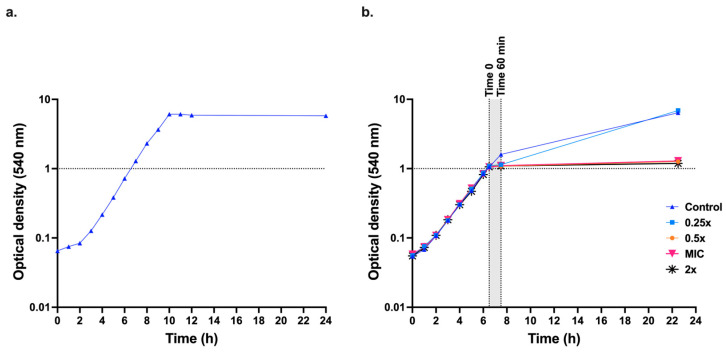
Effect of EOW on *C. albicans* SC5314 logarithmic phase YNBG cultures. (**a**) Growth curve for *C. albicans* SC5314 cells incubated in 50 mL YNBG at 37 °C with shaking (200 rpm). (**b**) Growth-MIC assay; when 250 mL YNBG cultures of *C. albicans* SC5314 reached an OD_540_ ≃1.0 (t = 0), EOW was added to achieve concentrations of 0 (control), 0.25×, 0.5×, 1×, or 2× MIC_90_ EOW-HOCl. OD_540_ measurements were taken 60 min and 16.5 h after t = 0.

Initially, *C. albicans* SC5314 cells were grown in 50 mL liquid medium at 37 °C with shaking at 200 rpm to ascertain whether the YNBG minimal medium lacking amino acids was suitable for growth of *C. albicans* (Figure 2a). These tests were necessary because typical yeast media contain sulphur containing compounds that quench the activity of EOW. YNBG medium was suitable for growing *C. albicans* cells, and it caused no quenching of EOW. The MIC_90_ of EOW-HOCl (named HOCl from now on) determined in YNBG in microtitre assays with an inoculum of 10^7^ cells/mL was 117.5 ± 7.32 µM (*n* = 16 replicates). The growth cutoff in HOCl MIC assays was sharp and MIC_50_ values were the same as MIC_90_ values. To determine the effect of HOCl on the growth of larger cultures, 250 mL YNBG cultures inoculated with an overnight YNBG culture to an OD_540_ ≃ 0.05 were incubated at 37 °C with shaking (200 rpm) until the OD_540_ = ~1.0. At this point (t = 0), cultures were exposed to 0.25×-, 0.5×-, 1×- or 2×- the HOCl MIC_90_ and the OD_540_ values were measured after 60 min and 16.5 h (Figure 2b). A culture that was not treated with HOCl served as the negative control (C). OD_540_ measurements revealed a continuation of growth for the untreated (control) culture at 60 min from t = 0. Among the HOCl-treated (T) cultures, the T0.25× treated cells showed a mild growth inhibition after 60 min; however, cells recovered and continued to grow. Culture dilutions plated at t = 60 min after T0.25× treatment revealed survivor colony counts similar to the control culture. OD_540_ measurements for the T0.5×-, T1×-, and T2×- MIC_90_ cultures revealed no further growth after the respective treatments. Survivor colony counts of cell dilutions of the T0.5×, T1×, and T2× cultures treated for 60 min were below the limit of detection. Further tests revealed a dramatically reduced (>10^4^ times) but still measurable survival rate (~10^3^ cfu/mL) for the T0.5× treated culture. A 60 min exposure time of late-logarithmic cells (grey shaded area, Figure 2b) was chosen to evaluate the effects of sub-growth-inhibitory HOCl concentrations (0.125×, 0.5× MIC_90_) on transcription.

### 3.2. Harvest of Cells After EOW Exposure and RNA Extraction and Purification

Cultures of *C. albicans* SC5314 (YNBG, 250 mL) were incubated at 37 °C with shaking until the OD_540_ = 1.0. Cultures were then either untreated (control) or treated for 60 min with 0.125×, or 0.5× MIC_90_ HOCl. Samples (100 mL) of triplicate cultures were harvested at t = 0 and t = 60 min after treatment and total RNA was extracted and purified as described in the methods section. All DNAse-treated RNA samples showed A260 nm/A280 nm absorption ratios of between 1.8 and 2.2, and the A260 nm/A230 nm ratios were 2 to 2.3 which indicated acceptable levels of sample purity for downstream qPCR and RNA-seq analysis.

Gel electrophoresis confirmed RNA integrity and the detection of equal amounts of RNA confirmed the purity of the RNA samples. The triplicate control, T0.125, and T0.5 RNA samples harvested at t = 60 min were analysed first by RT-qPCR and then sent for mRNA sequencing.

### 3.3. RT-qPCR Assays

The –RT and cDNA samples were subjected to RT-qPCR. Both housekeeping genes, *ACT1* and *TDH3*, were stably expressed. The expression levels (2^−^^∆∆Cq^) of the genes of interest were normalized to *TDH3* because it was expressed at higher levels than *ACT1* (lower C_q_ value).

There was no noticeable change to the mean C_q_ values for any of the candidate genes in response to 60 min exposure of cells to sub-growth inhibitory concentrations (0.125× or 0.5× MIC_90_) of HOCl (Table 2, Figure S3). The expression levels of *ACT1, CAT1, SOD1*, and *TRR1* (relative to *TDH3*) varied (Figure S3a,b), but treatment with either 0.125× or 0.5× MIC_90_ HOCl, did not significantly alter the expression of any of the genes relative to the control (Figure S3c,d, Table 3).

Thus, none of the candidate genes nor, as expected, any of the two housekeeping genes were up- or down-regulated in response to HOCl. In order to determine which genes did actually respond to HOCl treatment, we undertook a transcriptome analysis.

### 3.4. Transcriptome Response of C. albicans SC5314 to HOCl

#### 3.4.1. Filtered (Clean) Data and Assembly of Transcripts

Analysis of the mRNA sequencing data (Table S2) revealed mean total reads of 16,357,328, 18,578,325, and 16,453,547, for the control, T0.125, and the T0.5 groups, respectively. Of the total number of bases, 98% had quality scores higher than 20 (Q20); 94% (control), and 93% (T0.125, T0.5) had quality scores higher than 30 (Q30) (Q-Phred), indicating high sample purity. Of the total reads, 95%, 94%, and 93% from the control, T0.125, and T0.5 groups, respectively, aligned successfully to the reference genome.

#### 3.4.2. Differential Expression of *C. albicans* Genes Following Exposure to HOCl

**Figure 3:**
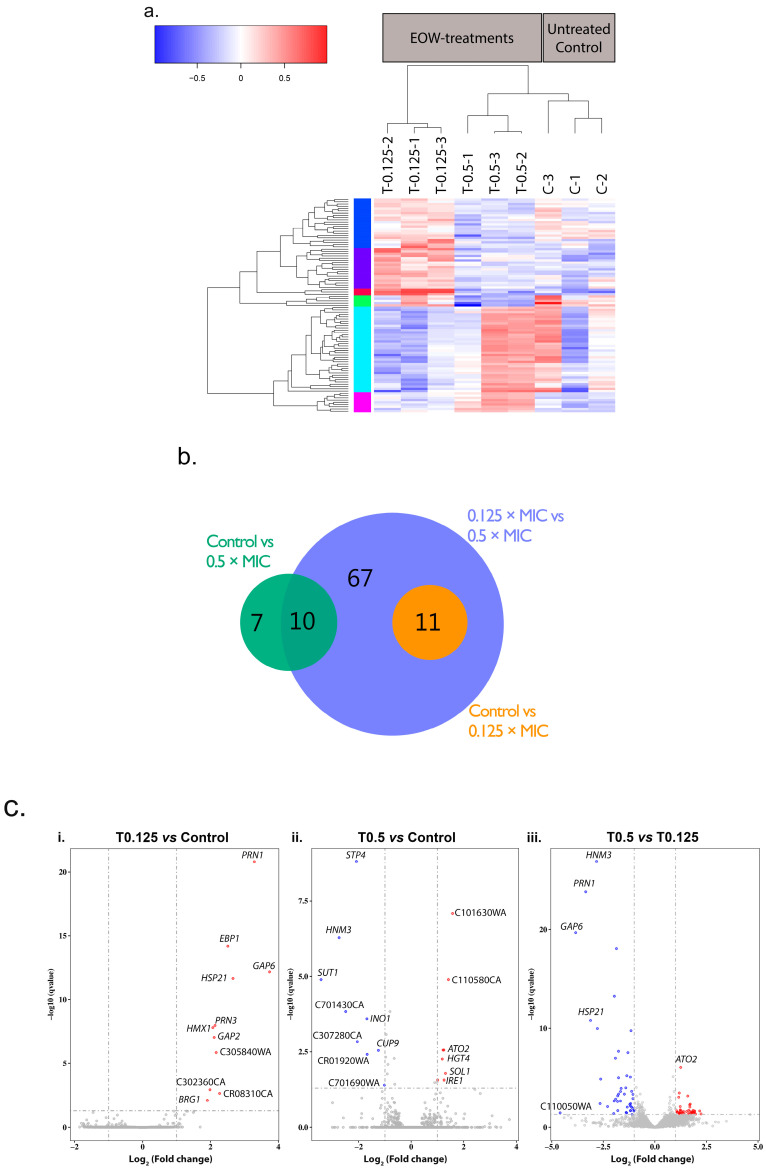
Comparison of gene expression in samples within and between treatment groups. (**a**) Heatmap dendrogram for the normalized mRNA expression levels (log_2_ of FPKMs) of the DEGs. Gene expression is relative to their combined average expression levels for each of the three biological replicates grown in the absence (60 min; C-1, -2, -3) or presence (60 min; T0.125-1, -2, -3; T0.5-1, -2, -3) of sub-growth inhibitory concentrations of HOCl. Colours of the vertical bar next to the *y*-axis of the dendrogram indicate different groups of transcripts with similar patterns of down- (blue) and/or up-regulation (red) between the various treatment groups. (**b**) Venn diagram showing the number of DEGs between the indicated pairs of treatment groups. (**c**) Genes differentially expressed in response to sub-growth-inhibitory HOCl. Volcano plots of the DEGs between the three pairs of treatment comparisons (**i**–**iii**) displaying the –log_10_ q value (*y*-axis) vs. log_2_ fold change (*x*-axis). The cut-off values (dotted grey lines) used to define significantly DEGs were q < 0.05 and a log_2_ fold change > |1|, i.e., larger than 2-fold (up- or down-regulated). Red data points—significantly upregulated genes; blue data points—significantly down regulated genes; grey data points—no significant change in expression.

A total of 95 genes were significantly differentially expressed (designated ‘differentially expressed genes’; DEGs) between the EOW treatment groups and the untreated control group (Figure 3). A heatmap dendrogram of the DEGs (Figure 3a) and a Principal Component Analysis (PCA) chart (Figure S4) show the relationship (clustering) between the triplicate repeats of EOW treated samples (T0.125-1, -2, -3; T0.5-1, -2, -3) and the control samples (C-1, C-2, C-3). One sample each from the treated (T0.5-1) and the untreated (C-1) groups was an outlier. The outliers could possibly be due to multiple freeze–thaw cycles of these total RNA samples. However, in the overall analyses, all triplicates were included in the log_2_ fold change calculations and in the downstream qPCR validation assays.

A Venn diagram (Figure 3b) and volcano plots (Figure 3c) showed that in the three comparisons of gene expression, 61 genes were upregulated and 55 were downregulated. When cells were treated with the lower HOCl concentration (T0.125), 11 genes were upregulated compared to the control. With the higher HOCl concentration (T0.5), eight genes were upregulated, and nine genes were downregulated. Forty-two and 46 genes were upregulated and downregulated, respectively, when expression in T0.5 cells was compared to expression in T0.125 cells. For all three comparisons there was a total of 116 DEGs; however there were 21 DEGs that were common between comparison groups (Figure 3b), hence there were 95 unique DEGs. GO annotations of the 56 most prominent GO terms enriched in the DEGs revealed that 33 genes are involved in molecular function, 17 are involved in cellular components, and 34 genes are involved in biological processes (Table S3).

#### 3.4.3. Transcriptional Response of *C. albicans* SC5314 Cells to Sub-Growth-Inhibitory Concentrations of HOCl

The transcriptional response of *C. albicans* SC5314 cells to EOW elicited two distinct concentration-based responses. With the 0.125× MIC_90_ HOCl treatment, 11 genes were upregulated with a log_2_ fold change in expression between 1.9 to 3.7 relative to the control (C) samples. A comparison of the mean FPKM values with those of the control and the T0.5 treatment groups demonstrated that these genes were specifically modulated in response to low concentrations of HOCl (0.125× MIC_90_), because the control and 0.5× MIC_90_-treated samples showed comparable expression levels (Table 4).

The biggest response of cells to 0.125× MIC_90_ was the upregulation of *GAP6*, *PRN1*, and *HSP21* (Figure 3c, Table 4; refer to Table S1 for gene characteristics), followed by two uncharacterized genes (CAALFM_CR08310CA and CAALFM_C305840WA), *EBP1*, *GAP2*, *PRN3*, *HMX1*, CAALFM_C302360CA, and *BRG1* Figure 3c; Table S1). Of note in the T0.125 comparison to the control cells (Table 4) is that no genes were significantly downregulated (Figure 3c); this is possibly because most of the downregulated genes were of very low abundance.

With the 0.5× MIC_90_ HOCl treatment (Group 2, Table 4), eight of the significantly upregulated genes (*ATO2*, *HGT4*, *SOL1*, *IRE1*, and the uncharacterized genes CAALFM_C101630WA, CAALFM_C110580CA, CAALFM_C305250CA, and CAALFM_CR06020WA) were at most 1.8 (log_2_) upregulated, suggesting that the cells had little chance to adapt to the higher (59 µM) HOCl concentration. The most significant upregulation was that of two uncharacterized genes (CAALFM_C101630WA and CAALFM_C110580CA), followed by *ATO2* (Figure 3c; Table S1). *ATO2* stands out as the gene with the most significantly upregulated transcript compared to the control and T0.125 cells (Table 4, Figure 3c). Of the nine significantly downregulated genes, three were genes of unknown function (CAALFM_C701690WA, CAALFM_CR01920WA, and CAALFM_C701430CA). *SUT1* was the most downregulated gene followed by *HNM3, STP4*, CAALFM_CR01920WA, *INO1*, and *CUP9* (Table 4).

When comparing the two treatments (Table 4), it is evident that the lower concentration and the higher concentration of HOCl elicited two completely different types of responses. *GAP6*, *PRN1*, and *HSP21* were the key genes upregulated in response to 0.125× MIC_90_ HOCl, based on their degree of upregulation and also their levels of expression (FPKM values). Some of the more significantly downregulated genes in response to the higher concentration of HOCl were involved in mitochondrial function and RNA or DNA processing. In summary, eleven genes were upregulated in response to 0.125× MIC_90_ HOCl treatment; the most notably differentially expressed genes were *GAP6*, *PRN1*, and *HSP21*. There were two uncharacterized genes (CAALFM_C101630WA, CAALFM_C110580CA) and *ATO2* that were specifically upregulated in response to 0.5× MIC_90_ HOCl. The most significant response to 0.5× MIC_90_ HOCl, however, was the downregulation of a number of genes, most notably *HNM3*, *INO1, SUT1*, and three uncharacterized genes (CAALFM_CR01920WA, CAALFM_C307280CA, CAALFM_C701430CA), which had relatively high expression levels in the control and T0.125 cells.

### 3.5. Confirmation of Gene Expression by RT-qPCR

**Figure 4:**
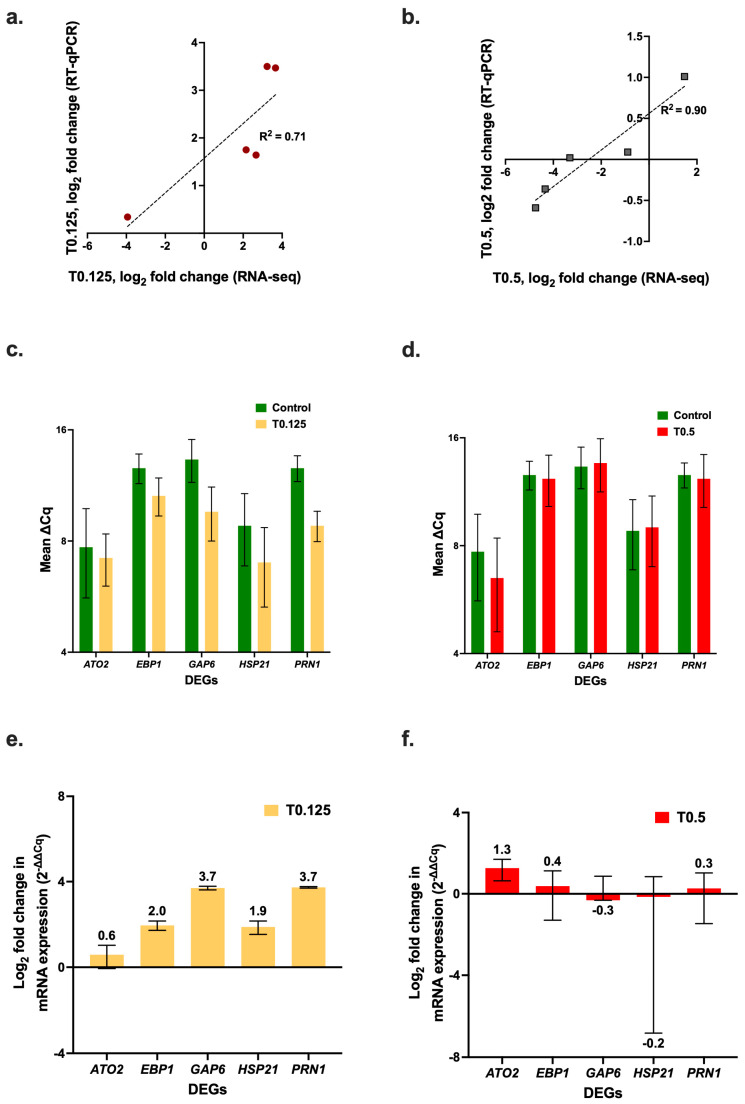
Quantification of *C. albicans* SC5314 gene expression levels in cells treated with sub-growth-inhibitory concentrations of HOCl. Correlation between the RT-qPCR and the RNA-seq log_2_-fold change values for the (**a**) 0.125× and (**b**) 0.5× MIC_90_ HOCl treatment samples. RT-qPCR quantification of *C. albicans* SC5314 *ATO1*, *EBP1*, *GAP6*, *HSP21*, and *PRN1* mRNA expression levels (∆C_q_) normalized with *TDH3* for the (**c**) 0.125× MIC_90_ and (**d**) 0.5× MIC_90_ HOCl treatment samples. Log_2_ fold change in expression (2^−∆∆Cq^) of logarithmic phase cells in response to 60 min exposure to (**e**) 0.125× MIC_90_ and (**f**) 0.5× MIC_90_ HOCl.

To validate the RNA-seq results, RT-qPCR assays were performed for five key DEGs (*ATO2*, *EBP1*, *GAP6*, *HSP21*, and *PRN1*) (Figure 4). The RT-qPCR results for the log_2_ fold change in expression of the DEGs showed a positive correlation with the RNA-seq results (Figure 4a, R^2^ = 0.71 for the T0.125 treatment, and Figure 4b, R^2^ = 0.90 for the T0.5 treatment). There was upregulation of all the DEGs that were selected for RT-qPCR, relative to the control with the 0.125× MIC_90_ HOCl treatment (Figure 4c); in contrast the 0.5× MIC_90_ HOCl treatment caused negligible changes in expression (Figure 4d). In general, there was greater upregulation by the T0.125 treatment (Figure 4e) than by the T0.5 treatment relative to the control; however, there was greater upregulation of *ATO2* in the T0.5 cells than in the T0.125 cells (Figure 4f).

## 4. Discussion

The hypothesis underpinning this study, based on previous research [25], was that treatment of *C. albicans* cells with sub-growth-inhibitory concentrations of EOW (HOCl) would elicit a significant change in expression levels of genes (*CAT1*, *SOD1*, *TRR1*) involved in the oxidative stress response pathway. However, there was no significant change in the expression of any of these genes in response to HOCl exposure, and hence, the research hypothesis was rejected. A recent study examining the effect of HOCl on *C. albicans* also found a minimal change in *SOD1* expression but reported increased expression of *CAT1* and *TRR1* as well as increased expression of *MXR1* and *SRX1*. The reason for the differences in gene expression in that study may be the different concentration of HOCl used (5 µM) and the different exposure time (15 min) [14]. In addition, the study did not state the source of HOCl or how the concentration was measured. In the present study, the responses of *C. albicans* cells to the lower (15 µM) and the higher (59 µM) concentrations of HOCl were distinctly different. Eleven genes of *C. albicans* cells exposed to 15 µM HOCl for 60 min were between 4-fold and 14-fold upregulated: *PRN1* and *PRN3* (proteins with similarity to pirins), *HSP21*, *GAP6*, and *GAP2* (two broad-specificity amino acid permeases), *EBP1* (an NADPH oxidoreductase), *HMX1* (a heme oxygenase), and two uncharacterized proteins (CAALFM_CR08310CA, CAALFM_C305840WA).

Two relatively recent reports [15,29] revealed an important function for Prn1 in the oxidative stress response of *C. albicans* to H_2_O_2_. An increase in Prn1 expression along with increases in the expression of Hsp21, Ebp1, and many other proteins involved in the oxidative stress response, protein folding, and proteasome-dependent catabolism were reported. In the more recent of the two studies [29], deletion of *PRN1* caused cells to accumulate increased levels of ROS when exposed to high concentrations of H_2_O_2_; a higher proportion of ∆*PRN1* knock-out cells showed signs of apoptosis and they also exhibited increased proteasomal activity when compared to wild-type control cells treated the same way. In *C. albicans*, the small heat shock protein Hsp21 enables cells to adapt to environmental stress (heat- and menadione-induced) by fine-tuning the homeostasis of intracellular stress protectants such as glycerol and trehalose via the activation of the Cek1 kinase pathway. Hsp21 was also found to be an important virulence factor of *C. albicans* [22]. Hsp21 is required to resist killing by human neutrophils; it is involved in regulating glycerol, glycogen, and trehalose homeostasis in response to elevated temperatures [22], and it also contributes to the response to ethanol-induced stress [48]. *GAP2* and *GAP6* are amino acid permeases and the uncharacterized gene, CAALFM_CR08310CA with a 6-fold upregulation, was assigned to processes involving alkanesulfonate catabolism with possible oxidoreductase and/or sulfonate dioxygenase activity. The upregulation of these genes may protect cells from the HOCl stress by removing potentially toxic proteins with HOCl modified sulphur containing amino acids and replenishing the cells with a fresh supply of amino acids from the surrounding environment.

A 60 min exposure of *C. albicans* cells to 59 µM HOCl mostly caused the downregulation of DEGs. Among the 17 DEGs, there was a significant downregulation of *SUT1* (a Zn_2_Cys6 transcription factor involved in sterol uptake), *HNM3* (a putative transporter), *STP4* (a C_2_H_2_ transcription factor), and *INO1* (inositol-1-phosphate synthase). There were only a few genes upregulated (<1.5 log_2_-fold) in response to 59 µM HOCl: *ATO2* (an ammonia exporter), *HGT4* (a high-affinity glucose transporter [26]), and *IRE1* (a protein kinase involved in the regulation of the unfolded protein response (UPR) [26]), indicating that cells struggled to cope with the stress at the higher HOCl concentration. This study used neutral-pH EOW with a pH value of 6.8 and was diluted 75-fold to achieve the higher (59 µM) HOCl concentration, thus it is unlikely that the vehicle for delivering the HOCl will have had a significant effect on gene expression.

Cells exposed to 15 µM HOCl showed a slight growth lag after the 60 min time-point, but recovered from the EOW-induced effects, as reflected in optical density measurements and plate counts that were similar to those of the no-treatment control (10^7^ cfu/mL). In contrast, exposure to 59 µM HOCl prevented further cell growth. Aliquots of these cultures from assay repeats revealed varied results, unlike those for the control and 0.125× MIC_90_ HOCl-treated cultures. The results ranged from significantly reduced cell counts (~10^3^ to 10^4^ cfu/mL) to no detectable live cell counts. Furthermore, the colony morphologies of cells grown in the presence of 0.5× MIC_90_ HOCl were also quite different from cells grown at the lower HOCl concentration; this raises the possibility that these cells were shutting down key cellular processes and were transitioning to a viable but nonculturable (VBNC) state, but this hypothesis requires verification. Literature on the effects of biocides on the VBNC state of fungi are limited. The VBNC state occurs in the phenol-degrading strain *Candida sp. LN1* (a strain closely related to *C. albicans* SC5314) as an adaption to redox stress [49]. Furthermore, the initial RT-qPCR experiments showed an ~2-fold downregulation of the *CAT1*, *SOD1*, and *TRR1* genes in response to 0.5× MIC_90_ HOCl (Figure S3d). This was also echoed in the RNA-seq transcriptomic response. It was previously reported that genes encoding proteins involved in the oxidative stress response (including superoxidase dismutase and thioredoxin reductase) were significantly downregulated when *Brettanomyces bruxellensis* yeast cells treated with 0.8 mg/L SO_2_ entered a VBNC state [50]. Similar adaptive responses may occur in *C. albicans* SC5314 cells that were exposed to 59 µM (0.5× MIC_90_) HOCl redox stress, but again this hypothesis requires verification. In another study [13], a 10 to 15 min exposure to HOCl concentrations, similar to those used in this research, significantly affected *S. cerevisiae* cell counts. A lack of instantaneous killing of *S. cerevisiae* cells was reported. Instead, the slow progressive loss of K^+^ from cells with increasing HOCl concentration indicated that the plasma membrane remained intact. It was proposed that the range of effects on metabolism meant that subsequent plating on rich medium may or may not give rise to cell replication and colony formation, hence, causing variable cell counts [13]. This may explain the variable cell counts observed for *C. albicans* cells exposed to 59 µM HOCl.

The UPR is an adaptive mechanism that is activated in response to the presence of unfolded or misfolded proteins in the ER [51]. The reliance of *C. albicans* on the ER-resident Ire1 protein for sensing ER stress and activating the UPR in response to antimicrobial (tunicamycin)-induced ER stress has been reported [51]. *C. albicans* cells respond to ER stress primarily by activating the conserved Ire1-Hac1-dependent UPR pathway and then by timely attenuation of the pathway once homeostasis is achieved by activation of the Ire-mediated *HOG1*-MAPK pathway [51,52]. Cell death in *S. cerevisiae* after exposure to 300 µM HOCl is mainly due to apoptosis that results in elevated levels of reactive oxygen species (ROS) and the formation of HOCl-modified proteins; HOCl lethality is mediated by the protease Kex1p [12]. The significant enrichment of the IRE-mediated UPR and the intrinsic apoptotic signalling pathway in response to ER stress that was triggered by exposure of *C. albicans* cells to 0.5× MIC_90_ of HOCl (59 µM) in the present study echoes these findings.

Previous studies suggested that *C. albicans* possesses additional mechanism(s) (absent in *S. cerevisiae*) by which H_2_O_2_ exposure triggers a superoxide stress response [53]. Such alternative mechanisms could possibly influence the adaptive response of *C. albicans* to HOCl. Taken together, our findings suggest that the stress response to sub-inhibitory concentrations of EOW triggers a unique stress response pathway that is quite distinct from the oxidative stress response pathway induced by H_2_O_2_ or menadione.

Limitations of stress-response studies include limited numbers of time points, variations of pH and growth media, and the use of varying concentrations of drugs between various research teams, all of which are likely to affect the results. Most *C. albicans* stress genes in response to H_2_O_2_ are induced within 10 to 30 min exposure times [17,18]. The choice of a 60 min time point is a possible limitation of the present study. Future time course studies should investigate a range of HOCl concentrations at additional time points including a five min exposure which is used in industry-standard disinfectant efficacy assays. Comparisons of global cellular responses among studies are limited by a lack of standard conditions for stress induction and transcription profile analysis; hence details of the activating process and measurement of the response varies [17]. The protocol used in the present study was limited by the quenching effects of RPMI-1640 on the EOW-HOCl [10], which is why we used YNBG without any amino acids to study the effects of HOCl on *C. albicans* cells.

The growing threat of opportunistic *C. albicans* infections in vulnerable individuals, including those residing in aged-care facilities, highlights the potential danger of *C. albicans* adaptive resistance to antimicrobial agents and to biocides. This study identified the genes that are possibly responsible for the stress response of *C. albicans* to sub-inhibitory HOCl concentrations. The role of these genes in biocide tolerance or resistance should be confirmed by gene deletions and by measuring their effects on the susceptibility of *C. albicans* cells to EOW and other stress inducers, including growth at elevated temperature (heat stress) or exposure to high salt (osmotic stress) or H_2_O_2_ (oxidative stress).

## 5. Conclusions

The present study identified Hsp21 and Prn1 as potentially important factors in protecting *C. albicans* cells against HOCl. A moderate sub-growth-inhibitory concentration (15 µM) of HOCl significantly upregulated *GAP6*, *HSP21*, and *PRN1*. A higher sub-growth-inhibitory concentration (59 µM) of HOCl induced mostly significant downregulation of *C. albicans* genes, reduced cell growth, and the upregulation of a few genes involved in key metabolic processes, including carbohydrate metabolism, ammonium transport, and the unfolded protein response pathway. The lethal action of HOCl on *C. albicans* cells may be due to ER protein unfolding events and apoptosis. Neutrophils produce high levels of myeloperoxidase, which converts H_2_O_2_ generated during the oxidative burst into the much more reactive HOCl. This study led to the hypothesis that Hsp21 and Prn1 are also key players in protecting *C. albicans* cells from neutrophil attack by the innate host immune system, but this requires validation.

## Figures and Tables

**Table 1:** DNA oligonucleotide primers used in the study.

Oligo Name	Sequence	Length of PCR Products (bp)
RT-qPCR primers designed for the pilot assays ^†^		
ACT1-416-for ^‡^ ACT1-493-rev ^‡^	CAGCTTTCTACGTTTCCATTCAAGC GTAAAGAGAAACCAGCGTAAATTGG	126
CAT1-706-for CAT1-778-rev	AGTAAATATACTGGCCAGAATAATG CTGGGTTACTTTTGGTTCCACATGC	121
SOD1-206-for SOD1-286-rev	CTGGTCCTCATTTCAATCCATTTGG ATAAATCTTGTTTGGTACCTTTAGC	129
TDH3-515-for ^‡^ TDH3-590-rev ^‡^	AACGATACTTTCGGTATTGAAGAAG ATGTTACCAGAAGCAGTTCTACCAC	122
TRR1-379-for TRR1-456-rev	ACTGGTGCCTCTGCCAAGAGAATGC AATCACAGCTAATGGGTTGTTTCTG	126
RT-qPCR primers designed for post-RNA-seq analyses ^†^		
ATO2-404-for ATO2-490-rev	TGACTGCATTGACATCTTACGG ATCCAATGGCATTTGGTAACTG	129
EBP1-349-for EBP1-439-rev	ATCAATGAAGCAATTCATGGCA CATCCCAATAAACTGCTGATGG	133
GAP6-1163-for GAP6-1248-rev	AATACGTTGACAGACAGGGAAG ACCAGACAAAGCGACTAACCAG	128
HSP21-280-for HSP21-366-rev	AAGACTACTGAAGAATCCGACA TTCTGTCTCTTGAGTAACAGTG	129
PRN1-337-for PRN1-421-rev	AATGCTGACGGTTCTCCAACTG CGTCATCTGTAACAACTTCTGG	127

^†^ Primer sequences were based on the *C. albicans* SC5314 reference genome sequence. ^‡^ Housekeeping gene. Abbreviations: for—forward; rev—reverse. Genes: *ACT1*—Actin; *ATO2*—Ammonia transport outward; *CAT1*—Catalase; *SOD1*—Superoxide dismutase; *TDH3*—Triose phosphate dehydrogenase; *TRR1*—Thioredoxin reductase; *EBP1*—Estrogen binding protein; *GAP6*—Broad-specificity amino acid permease; *HSP21*—Heat shock protein; *PRN1*—Protein with similarity to pirins.

**Table 2:** mRNA expression levels of *C. albicans* SC5314 cells exposed to sub-growth-inhibitory EOW-HOCl concentrations for 60 min.

Treatment	C_q_ (Mean)				
	*ACT1* ^†^	*TDH3* ^†^	*CAT1*	*SOD1*	*TRR1*
C	17.5	13.5	22.6	17.0	21.0
T0.125	17.6	13.8	22.6	17.5	21.3
T0.5	18.7	14.4	23.6	18.0	22.1

Mean C_q_ values of three independent experiments. C, untreated control; T: treatment with sub-growth-inhibitory EOW concentrations; 0.125, and 0.5 denote × MIC_90_ EOW-HOCl. ^†^ Housekeeping genes.

**Table 3:** *TDH3* normalised mean ∆∆ C_q_ values and log_2_ fold changes in mRNA expression levels relative to the *TDH3* normalised mean ∆∆ C_q_ values of the untreated control (C).

Gene	∆∆ C_q_		Log_2_ Fold Change	
	T0.125	T0.5	T0.125	T0.5
*ACT1*	−0.2	0.2	0.3	−0.2
*CAT1*	−0.4	0.1	0.4	−0.1
*SOD1*	0.2	0.0	−0.2	0.0
*TRR1*	−0.1	0.2	0.1	−0.2

**Table 4:** DEGs of *C. albicans* SC5314 in response to either lower (T0.125; group 1) or higher (T0.5; group 2) concentrations of HOCl.

Gene ID (CAALFM_)	Gene	FPKM (Mean ± SD)			Log_2_ Fold Change ^†^	Group *
		**C**	**T0.125**	**T0.5**		
**C503500WA**	***GAP6***	**2 ± 2**	**30 ± 6**	**3 ± 2**	**3.66**	**1**
**C105840WA**	***PRN1***	**9 ± 4**	**85 ± 23**	**10 ± 3**	**3.23**	**1**
**C204010CA**	***HSP21***	**100 ± 23**	**633 ± 197**	**105 ± 36**	**2.66**	1
**CR08310CA**		**14 ± 3**	**83 ± 19**	**14 ± 4**	**2.53**	**1**
**C305840WA**		**2 ± 1**	**10 ± 6**	**2 ± 0**	**2.25**	**1**
**C601180CA**	***EBP1***	**9 ± 4**	**40 ± 7**	**14 ± 1**	**2.15**	**1**
**C305580CA**	***GAP2***	**5 ± 1**	**20 ± 5**	**7 ± 1**	**2.08**	**1**
**C105870WA**	***PRN3***	**15 ± 6**	**61 ± 16**	**23 ± 10**	**2.07**	**1**
**C100350CA**	***HMX1***	**20 ± 6**	**84 ± 32**	**43 ± 5**	**2.05**	**1**
**C302360CA**		**5 ± 3**	**19 ± 8**	**6 ± 3**	**1.93**	**1**
**C105140WA**	***BRG1***	**2 ± 1**	**9 ± 2**	**3 ± 1**	**1.88**	**1**
C109250WA	*CRP1*	17 ± 6	54 ± 38	17 ± 2	1.67	1
C501800CA	*HIP1*	4 ± 3	12 ± 2	4 ± 1	1.58	1
C502790CA	*GAP1*	1 ± 1	3 ± 2	1 ± 1	1.58	1
C603700WA	*ALS1*	7 ± 2	18 ± 11	6 ± 3	1.36	1
C104650WA	*DUR3*	4 ± 2	10 ± 3	5 ± 3	1.32	1
C601070CA	*CIP1*	12 ± 4	30 ± 10	13 ± 1	1.32	1
C504180WA		11 ± 3	26 ± 8	13 ± 4	1.24	1
C504560CA	*CUP1*	2128 ± 1174	4578 ± 2522	2071 ± 42	1.11	1
C700350CA		114 ± 78	241 ± 54	131 ± 31	1.08	1
C209220WA	*DDR48*	121 ± 8	243 ± 96	128 ± 33	1.01	1
C400440CA	*OPT7*	14 ± 7	25 ± 4	11 ± 2	0.84	1
C104660WA	*DUR1,2*	8 ± 3	14 ± 3	7 ± 1	0.81	1
C405130CA	*ALD6*	43 ± 10	74 ± 19	37 ± 10	0.78	1
C104500WA	*ICL1*	21 ± 13	40 ± 6	13 ± 2	0.93	1
C110050WA		0 ± 0	2 ± 1	0.06 ± 0.1	ND	1
C113250WA		28 ± 22	18 ± 7	54 ± 27	−0.64	1
C106070WA		65 ± 41	41 ± 9	118 ± 60	−0.66	1
C300340WA		46 ± 13	29 ± 6	80 ± 29	−0.67	1
C406580WA	*CBF1*	45 ± 32	27 ± 12	85 ± 43	−0.74	1
C100700WA		102 ± 87	57 ± 31	176 ± 89	−0.84	1
CR04410WA		4 ± 2	2 ± 1	7 ± 5	−1.00	1
C306950WA		8 ± 5	4 ± 3	18 ± 9	−1.00	1
C601220CA		16 ± 13	8 ± 4	23 ± 9	−1.00	1
CR07760WA		84 ± 18	42 ± 18	116 ± 28	−1.00	1
C201740CA		71 ± 67	34 ± 11	121 ± 72	−1.06	1
CR04930WA	*PEX14*	16 ± 17	7 ±2	21 ± 10	−1.19	1
C401140CA		21 ± 17	9 ± 6	39 ± 19	−1.22	1
C502830WA		12 ± 12	5 ± 2	17 ± 9	−1.26	1
C204680WA	*TIM17*	70 ± 60	29 ± 11	115 ± 63	−1.27	1
CR06970CA		10 ± 8	4 ± 1	15 ± 9	−1.32	1
C502610CA	*RAD9*	5 ± 5	2 ± 1	6 ± 4	−1.32	1
C113550CA	*NUP49*	18 ± 19	7 ± 2	21 ± 10	−1.36	1
CR04830CA		3 ± 3	1 ± 1	6 ± 3	−1.58	1
C502990WA		12 ± 12	4 ± 3	18 ± 9	−1.58	1
CR09570WA		9 ± 8	3 ± 2	12 ± 7	−1.58	1
C102510WA		12 ± 9	4 ± 2	18 ± 10	−1.58	1
C206650CA		6 ± 8	2 ± 1	7 ± 4	−1.58	1
C201400CA	*ESC4*	6 ± 6	2 ± 1	6 ± 4	−1.58	1
C204780WA		12 ± 6	4 ± 2	15 ± 5	−1.58	1
C703630CA	*TIM9*	89 ± 100	29 ± 22	132 ± 71	−1.62	1
CR09720WA	*VMA22*	10 ± 6	3 ± 1	12 ± 6	−1.74	1
C201820CA		7 ± 8	2 ± 1	9 ± 4	−1.81	1
C204740CA		4 ± 3	1 ± 1	5 ± 1	−2.00	1
C301170WA		16 ± 15	4 ±3	17 ± 6	−2.00	1
C503030WA		4 ± 2	1 ± 1	5 ± 3	−2.00	1
CR03240CA		4 ± 4	1 ± 1	4 ± 1	−2.00	1
C502590CA		6 ± 6	1 ± 1	7 ± 4	−2.58	1
**C101630WA**		**67 ± 19**	**82 ± 9**	**232 ± 72**	**1.79**	**2**
**C110580CA**		**46 ± 14**	**52 ± 8**	**141 ± 29**	**1.62**	**2**
**C300930WA**	***ATO2***	**184 ± 23**	**195 ± 46**	**510 ± 19**	**1.48**	**2**
**C305250CA**		**7 ± 3**	**9 ± 2**	**18 ± 4**	**1.48**	**2**
**CR06020WA**		**8 ± 2**	**11 ± 3**	**22 ± 5**	**1.48**	**2**
**C304930CA**	***HGT4***	**5 ± 0**	**7 ± 1**	**14 ± 3**	**1.44**	**2**
C402640CA	*SEN2*	11 ± 3	10 ± 3	29 ± 12	1.40	2
**C303850CA**	***SOL1***	**18 ± 1**	**28 ± 7**	**47 ± 7**	**1.39**	**2**
**C107970CA**	***IRE1***	**10 ± 1**	**13 ± 2**	**24 ± 8**	**1.25**	**2**
C305290CA		7 ± 4	6 ± 2	15 ± 6	1.10	2
C402670WA		8 ± 3	6 ± 1	14 ± 3	0.81	2
C103650CA	*MRPL27*	78 ± 43	48 ± 21	134 ± 47	0.78	2
C603550CA		30± 8	18 ± 6	49 ± 23	0.71	2
CR06810WA	*HHT2*	571 ± 268	356 ± 130	902 ± 301	0.66	2
CR02140WA	*RSR1*	74 ± 34	42 ± 12	107 ± 48	0.53	2
C502460CA	*ECM331*	71 ± 11	103 ± 16	57 ± 8	−0.32	2
C102150WA	*GAL10*	8 ± 3	12 ± 3	6 ± 2	−0.42	2
C304480CA	*RAS2*	2 ± 1	3 ± 1	1 ± 0	−0.50	2
C112070CA		17 ± 0	21 ± 5	12 ± 1	−0.50	2
CR06660WA	*SEO1*	91 ± 44	112 ± 18	64 ± 19	−0.51	2
CR01630CA		12 ± 6	15 ± 3	8 ± 2	−0.58	2
CR02020CA	*OPT1*	9 ± 2	13 ± 3	6 ± 1	−0.58	2
CR04500CA		84 ± 28	116 ± 18	55 ± 12	−0.61	2
C703310WA		181 ± 74	216 ± 72	117 ± 11	−0.63	2
C209950WA	*FCY21*	168 ± 43	175 ± 56	99 ± 6	−0.76	2
**C701690WA**		**64 ± 10**	**48 ± 6**	**37 ± 7**	**−0.80**	**2**
CR01910CA		170 ± 24	161 ± 23	96 ± 41	−0.82	2
CR01930CA	*BIO2*	803 ± 166	848 ± 28	445 ± 32	−0.85	2
C106610CA	*HAK1*	27 ± 15	32 ± 3	14 ± 2	−0.95	2
**C702040CA**	***CUP9***	**97 ± 23**	**79 ± 10**	**47 ± 6**	**−1.04**	2
**CR01920WA**		**100 ± 16**	**116 ± 6**	**37 ± 18**	**−1.43**	**2**
**CR10100CA**	***INO1***	**1718 ± 508**	**2155 ± 336**	**639 ± 70**	**−1.43**	**2**
**C307280CA**		**168 ± 52**	**127 ± 61**	**45 ± 6**	**−1.90**	**2**
**C203220CA**	***STP4***	**104 ± 85**	**72 ± 35**	**27 ± 5**	**−1.93**	**2**
**C701430CA**		**102 ± 80**	**57 ± 30**	**20 ± 8**	**−2.35**	**2**
**CR01900CA**	***HNM3***	**115 ± 19**	**147 ± 28**	**21 ± 13**	**−2.43**	**2**
**C503130WA**	***SUT1***	**82 ± 85**	**33 ± 29**	**8 ± 4**	**−3.29**	**2**

* Group 1, DEGs due to exposure to the lower concentration of HOCl (0.125× MIC_90_), and Group 2, DEGs due to exposure to the higher concentration of HOCl (0.5× MIC_90_). ^†^ Log_2_ fold change refers to the up- or downregulation of the gene relative to the untreated control. Genes in **bold** were significantly up- or down-regulated. ND, Log_2_ fold change could not be determined.

## Data Availability

The data presented in this study are available on request from the corresponding author.

## Supplementary Materials

The following supporting information can be downloaded at: https://www.mdpi.com/article/10.3390/jof11110761/s1.

## Abbreviations

The following abbreviations are used in this manuscript:

cDNA	complementaryDNA
CGD	Candida Genome Database
C_q_	Quantification Cycle (threshold cycle)
DAG	Directed Acrylic Graph
DEGs	Differentially Expressed Genes
DEU	Differential Exon Usage
DNA	Deoxyribonucleic Acid
EOW	Electrolysed Oxidising Water
ER	Endoplasmic Reticulum
EUCAST	European Committee on Antimicrobial Susceptibility Testing
FPKM	Fragments Per Kilobase per Million reads
GO	Gene Ontology Consortium Database
KEGG	Kyoto Encyclopedia of Genes and Genomes
MIC	Minimum Inhibitory Concentration
MOPS	3-(N-morpholino)propanesulfonic acid
mRNA	messengerRNA
NGS	Next Generation Sequencing
OD	Optical Density
PCA	Principal Component Analysis
RNA-seq	Ribonucleic Acid-sequencing
ROS	Reactive Oxygen Species
RPKM	Reads Per Kilobase of transcript per Million reads
RPMI	Rosewell Parks Memorial Institute
RT	Reverse Transcriptase
RT-qPCR	Reverse Transcription-Quantitative Polymerase Chain Reaction
RTU	Ready-to-Use
SDA	Sabouraud’s Dextrose Agar
UPR	Unfolded Protein Response
UV–Vis	UltaViolet–Visible
VBNC	Viable But Non-Culturable
YNBG	Yeast Nitrogen Base Glucose
