# Supplementary material for: Molecular Mechanism of Action of HOCl from Neutral-pH Electrolysed Oxidising Water Against Candida albicans
Source: J Fungi (Basel). 2025 Oct 23;11(11):761. doi: 10.3390/jof11110761 (PMC12653471; doi:10.3390/jof11110761)
Supplement: Supplementary file 1 [file jof-11-00761-s001.zip › JoF-3872786-Supplementary Materials.pdf]

Journal of Fungi

MDPI

# Supplementary Materials

## Supplementary Methods

*Workflow for mRNA-sequencing: mRNA library construction and sequencing*

The experimental workflow (Figure S2) included the following steps: total RNA quality control (QC), mRNA purification, cDNA synthesis, cDNA purification and amplification, and cDNA library QC and quantitation, using the library construction kit (VAHTS® Universal V8 RNA-seq Library Prep Kit for Illumina, Vazyme, Nanjing Vazyme Biotech Co., Ltd., Nanjing, PRC). All steps were monitored to ensure the accuracy and reliability of the results. For each sample, 1 μg total RNA was used for library preparation. Poly(A) mRNA isolation was performed using Oligo(dT) beads (VAHTS mRNA Capture Beads, Vazyme, Nanjing, PRC). mRNA fragmentation was achieved using divalent cations and the manufacturer-recommended PCR protocol (94°C for 8 min). First strand cDNA and second strand cDNA were synthesized as per the manufacturer's protocol. The double-stranded cDNA was purified, treated to repair both ends and to add dA-tails, followed by a T-A ligation to add library-specific adaptors to both ends. Size selection of adaptor-ligated DNA was then performed using DNA Clean Beads (VAHTS Clean Beads, Vazyme, Nanjing, PRC). Each sample was then amplified by PCR using random primers (VAHTS i5 PCR Primer and VAHTS i7 PCR Primer, Vazyme, Nanjing, PRC). PCR products (1 μL) were validated using an Agilent DNA 1000 chip and digitally analyzed (Agilent Technologies 2100 Bioanalyzer, Santa Clara, CA, USA). Then libraries with the different adaptors were multiplexed and loaded on an Illumina Novaseq6000 (Illumina, San Diego, CA, USA) instrument for sequencing using a 2 × 150 paired end (PE) configuration according to the manufacturer's instructions. The high-throughput sequencing was performed after mixing sample libraries based on their effective concentration and the required sequencing data volume.

**Table S1:** Details of *C. albicans* SC5314 genes of interest in this study.

| Gene ID | Gene name | ORF (bp) | Gene description† |
|---|---|---|---|
| C113700WA | *ACT1* | 1789 | Actin housekeeping gene |
| C300930WA | *ATO2* | 798 | Ammonia transporter; spider biofilm induced |
| C106810WA | *CAT1* | 1458 | Catalase; resistance to oxidative stress, neutrophils, peroxide; Spider biofilm induced |
| C601180CA | *EBP1* | 1224 | Estrogen-binding protein; NADPH oxidoreductase; induced by oxidative, weak acid stress; rat catheter biofilm induced |
| C503500WA | *GAP6* | 1707 | Broad-specificity amino acid permease; rat catheter biofilm induced |
| C304930CA | *HGT4* | 2247 | Glucose and galactose sensor; 20-member glucose transporter family; Spider biofilm induced |
| C100350CA | *HMX1* | 876 | Heme oxygenase; repressed by Efg1; upregulated by Rim101 at pH 8; Spider and flow model biofilm induced |
| CR01900CA | *HNM3* | 1497 | Putative transporter; possibly essential, Spider biofilm induced |
| C204010CA | *HSP21* | 570 | Small heat shock protein; role in stress response and virulence; Spider biofilm induced |
| CR10100CA | *INO1* | 1563 | Inositol-1-phosphate synthase; phospholipid biosynthesis of PI; Spider biofilm repressed |
| C107970CA | *IRE1* | 3672 | Protein kinase involved in regulation of unfolded protein response; mutant is hypersensitive to caspofungin; Spider biofilm induced |
| C105840WA | *PRN1* | 1038 | Protein with similarity to pirins; induced by benomyl and in response to alpha pheromone in SpiderM medium; transcript induced by Mnl1 in weak acid stress; rat catheter and Spider biofilm induced |
| C402320CA | *SOD1* | 710 | Cytosolic copper- and zinc-containing superoxide dismutase; protection from oxidative stress; required for full virulence; alkaline induced by Rim101; |
| C203220CA | *STP4* | 1131 | C2H2 transcription factor; rat catheter and Spider biofilm induced |
| C503130WA | *SUT1* | 1071 | Zinc cluster transcription factor involved in sterol uptake |
| C306870WA | *TDH3* | 1008 | glyceraldehyde-3-phosphate dehydrogenase housekeeping gene |
| C502710WA | *TRR1* | 963 | Thioredoxin reductase; induced by nitric oxide, peroxide; oxidative stress-induce via Cap1; induced by human neutrophils |

†*C. albicans* SC5314 gene details are from the *Candida* Genome Database [26].

**Table S2:** Filtered data statistics and data alignment statistics for the sequenced samples.

| Sample† | Total reads‡ | Number of bases | Q20§ (%) | Q30§ (%) | Total mapped¶ | Multiple mapped# | Uniquely mapped†† |
|---|---|---|---|---|---|---|---|
| C-1 | 14,172,852 | 2,046,101,850 | 97.80 | 93.69 | 13,419,228 (94.68%) | 749,704 (5.29%) | 12,669,524 (89.4%) |
| C-2 | 18,069,344 | 2,637,240,315 | 97.92 | 93.89 | 17,062,386 (94.43%) | 1,027,602 (5.69%) | 16,034,784 (88.74%) |
| C-3 | 16,829,788 | 2,457,246,960 | 97.91 | 93.83 | 15,885,737 (94.39%) | 746,659 (4.44%) | 15,139,078 (89.95%) |
| T0.125-1 | 15,670,526 | 2,258,528,435 | 96.96 | 91.98 | 14,622,739 (93.31%) | 817,745 (5.22%) | 13,804,994 (88.1%) |
| T0.125-2 | 19,305,444 | 2,762,328,172 | 97.99 | 94.14 | 18,226,126 (94.41%) | 1,011,682 (5.24%) | 17,214,444 (89.17%) |
| T0.125-3 | 20,759,004 | 3,060,836,007 | 97.86 | 93.68 | 19,480,408 (93.84%) | 999,118 (4.81%) | 18,481,290 (89.03%) |
| T0.5-1 | 13,459,748 | 1,969,353,442 | 97.69 | 93.38 | 12,582,153 (93.48%) | 678,568 (5.04%) | 11,903,585 (88.44%) |
| T0.5-2 | 18,184,600 | 2,636,307,635 | 97.78 | 93.51 | 16,992,147 (93.44%) | 839,062 (4.61%) | 16,153,085 (88.83%) |
| T0.5-3 | 17,716,292 | 2,600,998,872 | 97.76 | 93.41 | 16,536,543 (93.34%) | 727,764 (4.108%) | 15,808,779 (89.23%) |

†Sample: All samples (n = 3 per group) harvested at time 60 min from $OD_{540}$ = 1.0. C, Control (untreated); T0.125, treated with 0.125× $MIC_{90}$ HOCl; T0.5, treated with 0.5× $MIC_{90}$ HOCl.

‡ Total reads: Number of the total reads that passed the filtering step.

§Q20, Q30: The percentage of bases with quality scores (Q-phred) higher than 20 or 30.

¶Total mapped: Number of reads that were successfully aligned to the reference genome. In general, the percentage mapped should be >70%, given that there is no contamination, and an appropriate reference was selected.

#Multiple mapped: The number of sequences with multiple alignment positions on the reference genome. This value is generally <10%.

††Uniquely mapped: number of sequences with only one alignment position on the reference genome.

**Table S3**: Gene ontology (GO) annotations of the GO terms describing DEG function

| GO Term | Category | DEGs | | |
|---|---|---|---|---|
| | | **Control *vs* T0.125** | **Control *vs* T0.5** | **T0.125 *vs* T0.5** |
| amino acid transmembrane transporter activity | **Molecular function (28)** | *GAP2*, *GAP6* | | *GAP1*, *GAP2*, *GAP6*, *HIP1* |
| sequence–specific DNA binding | | *BRG1* | | |
| FMN binding | | *EBP1* | | |
| pentaerythritol trinitrate reductase activity | | *EBP1* | | |
| NADPH dehydrogenase activity | | *EBP1* | | |
| oxidoreductase activity, acting on paired donors, with incorporation or reduction of molecular oxygen, 2–oxoglutarate as one donor, and incorporation of one atom each of oxygen into both donors | | CAALFM_CR08310CA | | |
| sulfonate dioxygenase activity | | CAALFM_CR08310CA | | |
| endoribonuclease activity | | | *IRE1* | |
| sugar: proton symporter activity | | | *HGT4* | |
| ammonium transmembrane transporter activity | | | *ATO2* | |
| acetate transmembrane transporter activity | | | *ATO2* | |
| adenosylmethionine–8–amino–7–oxononanoate transaminase activity | | | CAALFM_CR01920WA | |
| inositol–3–phosphate synthase activity | | | *INO1* | *INO1* |
| malonate–semialdehyde dehydrogenase (acetylating, NAD+) activity | | | | *ALD6* |
| malonate–semialdehyde dehydrogenase (acetylating) activity | | | | *ALD6* |
| allophanate hydrolase activity | | | | *DUR1%2C2* |
| urea carboxylase activity | | | | *DUR1%2C2* |
| fibronectin binding | | | | *ALS1* |
| laminin binding | | | | *ALS1* |
| urea transmembrane transporter activity | | | | *DUR3* |
| putrescine transmembrane transporter activity | | | | *DUR3* |
| UDP–glucose 4–epimerase activity | | | | *GAL10* |
| aldose 1–epimerase activity | | | | *GAL10* |
| allantoate transmembrane transporter activity | | | | *SEO1* |
| arylsulfatase activity | | CAALFM_C302360CA | | CAALFM_C302360CA |
| isocitrate lyase activity | | | | *ICL1* |
| methylisocitrate lyase activity | | | | *ICL1* |
| methylmalonate–semialdehyde dehydrogenase (acylating) activity | | | | *ALD6* |

| GO Term | Category | DEGs | | |
|---|---|---|---|---|
| | | Control *vs* T0.125 | Control *vs* T0.5 | T0.125 *vs* T0.5 |
| plasma membrane | **Cellular component (4)** | | | *ALS1*, *ATO2*, *DUR3*, *ECM331*, *FCY21*, *GAP1*, *GAP2*, *GAP6*, *HIP1*, *RSR1* |
| cell surface | | | | *ALS1*, *ECM331*, *ENO1*, *GAP1* |
| mitochondrial small ribosomal subunit | | | | CAALFM_C106070WA, CAALFM_CR07760WA |
| IRE1–TRAF2–ASK1 complex | | | *IRE1* | |
| amino acid transmembrane transport | **Biological process (25)** | *GAP2*, *GAP6* | | *GAP1*, *GAP2*, *GAP6*, *HIP1* |
| alkanesulfonate catabolic process | | CAALFM_CR08310CA | | |
| sulfur compound catabolic process | | CAALFM_CR08310CA | | |
| sulfolipid metabolic process | | CAALFM_C302360CA | | CAALFM_C302360CA |
| carbohydrate transport | | | *HGT4* | |
| phospholipid biosynthetic process | | | *INO1* | |
| nitrogen utilization | | | *ATO2* | |
| ammonium transport | | | *ATO2* | |
| endoplasmic reticulum unfolded protein response | | | *IRE1* | |
| glucose mediated signaling pathway | | | *HGT4* | |
| response to endoplasmic reticulum stress | | | *IRE1* | |
| biotin biosynthetic process | | | CAALFM_CR01920WA | *BIO2*, CAALFM_CR01920WA |
| protein localization to Golgi apparatus | | | *IRE1* | |
| inositol metabolic process | | | *IRE1* | |
| inositol biosynthetic process | | | *INO1* | *INO1* |
| detection of glucose | | | *HGT4* | |
| intrinsic apoptotic signaling pathway in response to endoplasmic reticulum stress | | | *IRE1* | |
| IRE1–mediated unfolded protein response | | | *IRE1* | |
| protein trans–autophosphorylation | | | *IRE1* | |
| thymine metabolic process | | | | *ALD6* |
| valine metabolic process | | | | *ALD6* |
| putrescine transport | | | | *DUR3* |
| urea transport | | | | *DUR3* |
| positive regulation of flocculation | | | | *ALS1* |
| urea catabolic process | | | | *DUR1%2C2* |

Gene ontology (GO) annotations of the 57 most prominent GO terms enriched in the DEGs grouped into three main GO categories: molecular function (28), cellular component (4), and biological process (25).

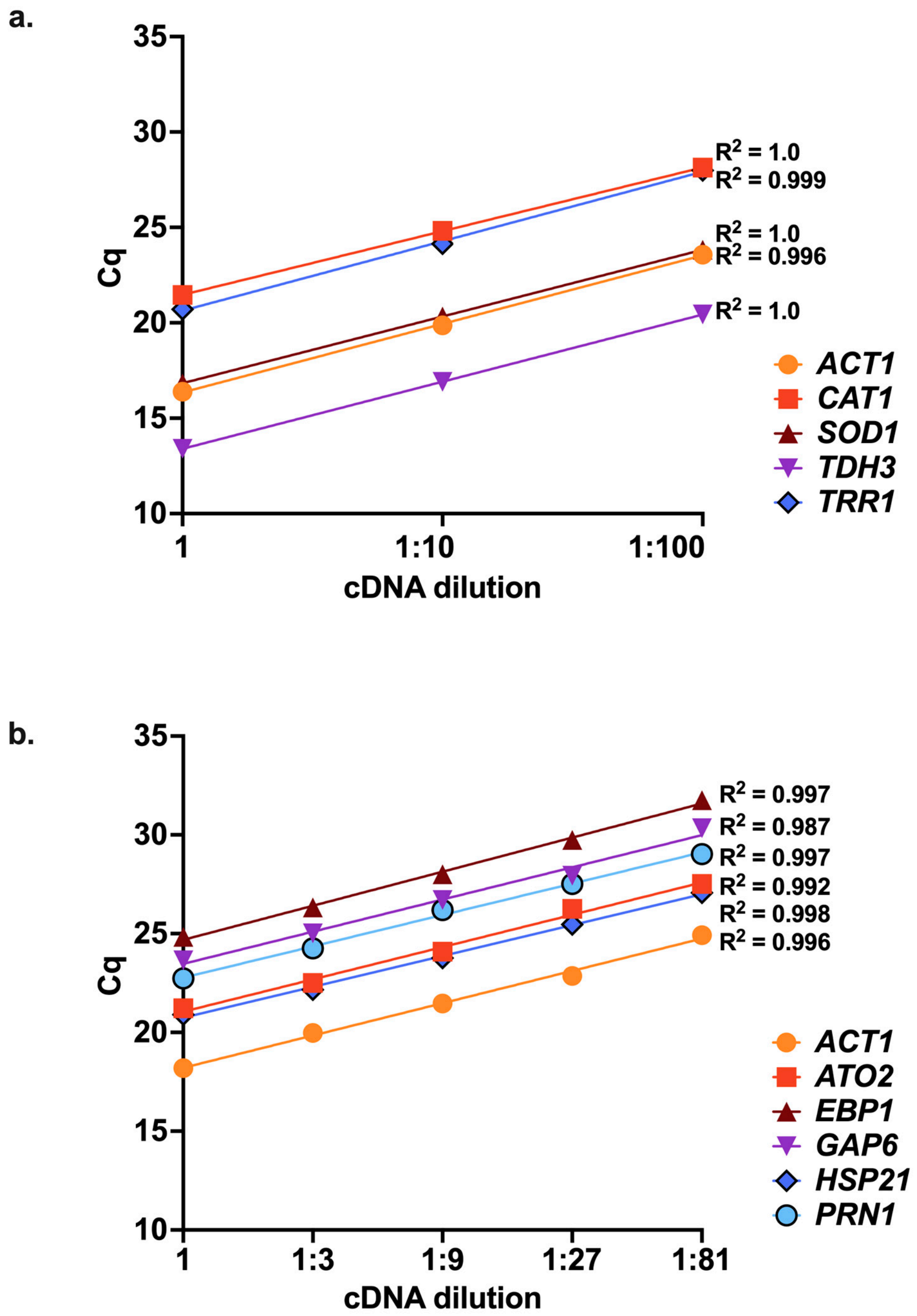


**Figure S1**: Representative correlation plots between $C_q$ values and cDNA dilutions to confirm amplification efficiencies from: (**a**) pilot RT-qPCR assays (C0 sample), and (**b**) validation RT-qPCR assays performed post-transcriptomic analyses (T0.125-3).

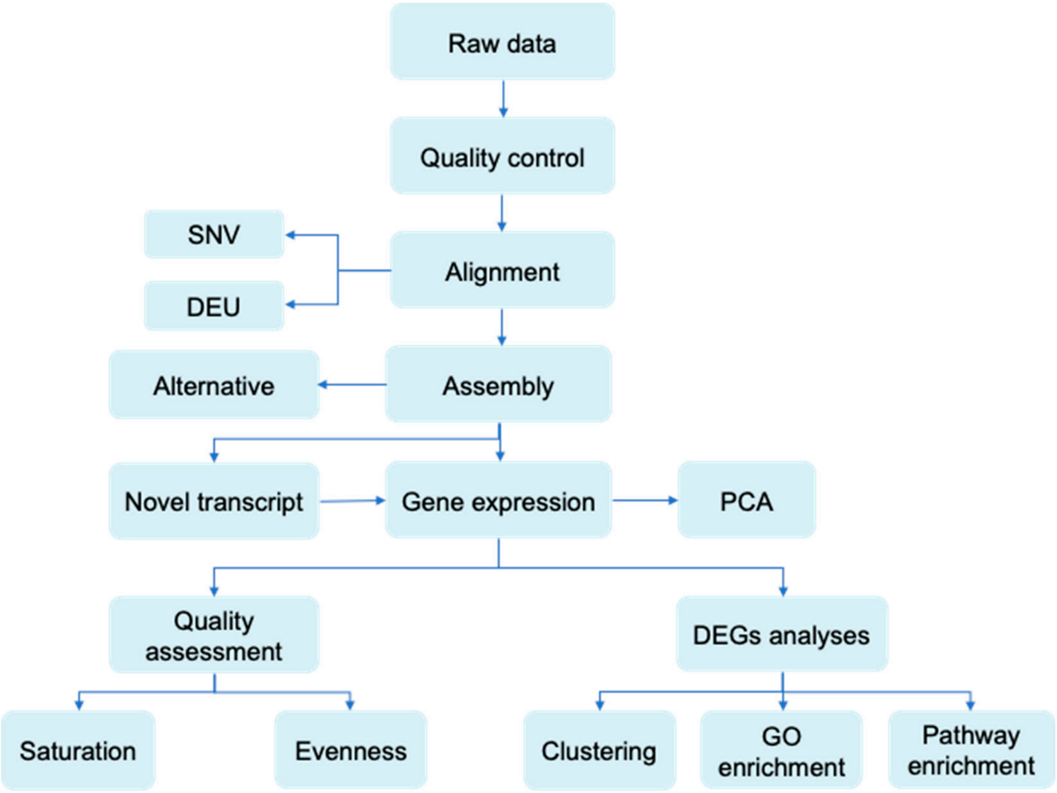


**Figure S2**: Bioinformatics workflow pipeline performed by Genewiz® Biotechnology Co. Ltd., Azenta Life Sciences, Suzhou, PRC. Abbreviations: DEU–Differential Exon Usage; DEGs–Differentially Expressed Genes; GO–Gene Ontology; PCA–Principal Component Analysis; SNV–Single Nucleotide Variant.

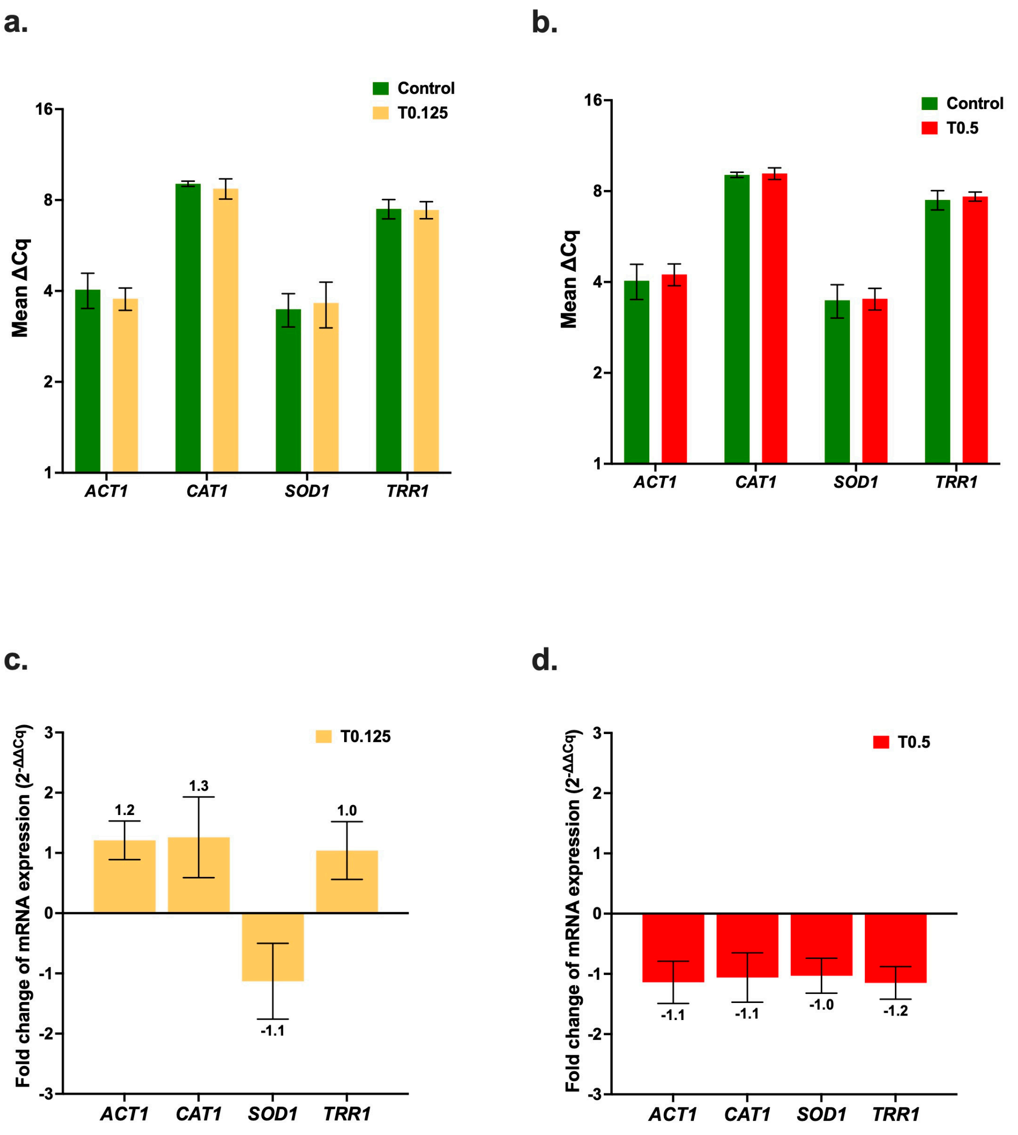


**Figure S3:** Effect of sub-MIC90 HOCl exposure on *C. albicans* SC5314 *ACT1, CAT1, SOD1* and *TRR1* expression levels. Pilot RT-qPCR quantification of n=3 biological replicates from Control, T0.125 and T0.5 samples harvested at the 60 min timepoint. Mean *C. albicans* SC5314 *ACT1, CAT1, SOD1, TRR1* mRNA expression levels normalized to *TDH3* expression ($\Delta C_q$) for the: (**a**) 0.125× MIC90 HOCl and Control samples; and (**b**) 0.5× MIC90 HOCl and Control samples. (**c**) The mean fold change in mRNA expression ($2^{-\Delta\Delta Cq}$) of cells in response to a 60 min treatment with 0.125×- or (**d**) 0.5×-MIC90 HOCl, relative to expression in the control group.

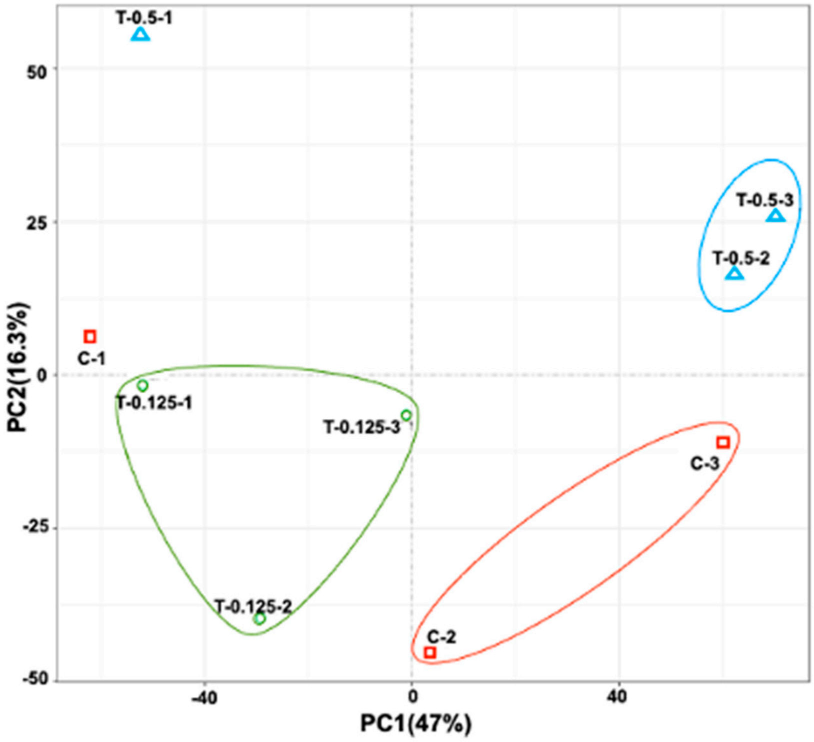


**Figure S4**: Principal Component Analyses (PCA) chart showing the relationships (clustering) between the samples.
